# Evaluation *in vitro* of the virulence of two entomopathogenic heterorhabditid nematodes in the control of *Stomoxys calcitrans* (Diptera: Muscidae) larvae in byproducts of the sugar and alcohol industry

**DOI:** 10.1590/S1984-29612023024

**Published:** 2023-04-28

**Authors:** Américo de Castro Monteiro, Luís Carlos de Souza Rodrigues Leal, João Luiz Lopes Monteiro, Melissa Carvalho Machado do Couto Chambarelli, Avelino José Bittencourt

**Affiliations:** 1 Programa de Pós-graduação em Ciências Veterinárias, Universidade Federal Rural do Rio de Janeiro - UFRRJ, Seropédica, RJ, Brasil; 2 Programa de Pós-graduação em Agronomia, Universidade Federal de Roraima - UFRR, Boa Vista, RR, Brasil; 3 Departamento de Parasitologia Animal, Universidade Federal Rural do Rio de Janeiro - UFRRJ, Seropédica, RJ, Brasil; 4 Departamento de Medicina e Cirurgia Veterinária, Universidade Federal Rural do Rio de Janeiro - UFRRJ, Seropédica, RJ, Brasil

**Keywords:** Heterorhabditis, stable fly, sugar and alcohol byproducts, biological control, Heterorhabditis, mosca-dos-estábulos, subprodutos sucroalcooleiros, controle biológico

## Abstract

*Stomoxys calcitrans* causes losses to livestock, mainly to cattle. This study aimed to determine the pathogenic potential of *Heterorhabditis bacteriophora* HP88 and *H. baujardi* LPP7 against *S. calcitrans* larvae after being exposed to byproducts of the sugar and alcohol industry. The efficacy of EPNs on stable fly larvae was evaluated in bioassays with vinasse at three temperatures (16, 25 and 35 °C) and concentrations (0, 50 and 100%), as well as in relation to larva age (4, 6 and 8 days) in filter cake and EPNs concentration (100, 300 and 500 IJs/larva) in sugarcane bagasse. *H. bacteriophora* showed higher efficacy than *H. baujardi* at all temperatures. Vinasse did not have a negative effect on the virulence of *H. bacteriophora*. The age of fly larvae did not affect the mortality rates caused by the EPNs. In bagasse, *H. bacteriophora* presented higher mortality rates than the control group. It is concluded that EPNs can be a potential component in integrated strategies of stable fly control and outbreak prevention in areas of sugar and alcohol production.

## Resumo

*Stomoxys calcitrans* causa perdas à agropecuária, principalmente na bovinocultura. Este estudo objetivou verificar o potencial patogênico de *Heterorhabditis bacteriophora* HP88 e *H. baujardi* LPP7 para larvas de *S. calcitrans*, após serem expostos a subprodutos da indústria sucroalcooleira. A eficácia dos NEPs sobre larvas da mosca-dos-estábulos foi avaliada em bioensaios com vinhaça em três temperaturas (16, 25 e 35 °C) e concentrações (0, 50 e 100%), bem como em relação à idade da larva (4, 6 e 8 dias) em torta de filtro e concentração de NEPs (100, 300 e 500 JIs/larva) em bagaço de cana-de-açúcar. *H. bacteriophora* apresentou maior eficácia do que *H. baujardi* em todas as temperaturas. O vinhoto não apresentou efeito negativo sobre a virulência de *H. bacteriophora*. A idade das larvas não influenciou no aumento ou diminuição da mortalidade dos NEPs. Em bagaço de cana, *H. bacteriophora* apresentou taxas de mortalidade superiores à do grupo controle. Conclui-se que os NEPs podem ser um potencial componente em estratégias de controle integrado e prevenção de surtos da mosca-dos-estábulos, em áreas de produção de açúcar e álcool.

## Introduction

*Stomoxys calcitrans*, commonly called the stable fly, is a hematophagous dipteran that parasitizes several animal species, including cattle, goats, equines, pigs, and even humans. Its geographical distribution is wide, and its greatest population increase occurs during the hottest months of the year (Bittencourt & Moya-Borja, 2000). Parasitism by the stable fly poses a serious problem in raising livestock in several regions of Brazil. The country has suffered estimated economic losses of USD 335.5 million (Grisi et al., 2014). Several materials can be used to develop immature stages of *S. calcitrans*, particularly fermenting organic matter (Lysyk et al., 1999). The cultivation and generation of byproducts of the sugarcane industry provide a favorable environment for the proliferation of *S. calcitrans*, given the abundance of substrates such as straw and sugarcane bagasse, filter cake and vinasse, which, together, favor the occurrence of fly population outbreaks. Barros et al. (2010) reported an outbreak of stable flies in the state of Mato Grosso do Sul, where the authors associated the large numbers of flies with vinasse used in the irrigation of sugarcane plants.

A class of agents with potential for use in biological control are entomopathogenic nematodes (EPNs), which are obligate parasites of soil-dwelling insects (Kaya & Gaugler, 1993). The importance of EPN as biological control agents has been revealed in the results of several studies published in recent decades, which describe their pathogenic effect on various types of pests. However, in Brazil, the use of EPN has been studied mainly in agriculture, and the number of researches evaluating its action on pests of veterinary importance is still scanty. In response to the heightened resistance of insect pests to chemical pesticides (Barros et al., 2019), mainly due to the excessive use, research has focused on different microorganisms over the years, seeking to identify their pathogenic potential on different parasites of economic importance, and their feasibility for use in biological control or application in integrated control (chemical and biological).

The purpose of this study was to determine the effectiveness of the EPNs *Heterorhabditis bacteriophora* HP88 and *H. baujardi* LPP7 against *S. calcitrans* larvae after being exposed to byproducts of the sugar and alcohol industry, and the response of EPNs when exposed to vinasse at different temperatures (investigate whether high vinasse temperatures can cause any risk to EPNs) and their action on *S. calcitrans* larvae of different ages.

## Material and Methods

The *S. calcitrans* colony used in this study was raised on a laboratory benchtop (27±1° C and 70-80% relative humidity - RH) using the methods described by Mello (1989) and Moraes (2007). The EPNs colonies followed the method described by Lindegren et al. (1993), and it was maintained and multiplied via *in vivo* multiplication in *Galleria mellonella* (Lepidoptera: Pyralidae). Infective juveniles (IJs) were stored in a Biochemical Oxygen Demand (BOD) temperature-controlled incubator (Eletrolab®, model EL 202/4) at 16 ± 1° C and 70-80% RH in a 40 mL cell culture flask for less than 7 days. To calculate the levels used in this study, IJs were counted in twelve 10 μL aliquots taken from an aqueous EPNs suspension. After counting the IJs in the 12 aliquots, the highest and lowest number of EPNs/aliquot were discarded and the average number of IJs in the remaining 10 aliquots was calculated. Based on this calculation, the concentration of the suspensions was adjusted to IJs/mL (Taylor et al., 1998).

### Bioassay I

#### Efficacy of EPNs against *S. calcitrans* larvae in vinasse

EPNs were quantified and adjusted to a concentration of 2000 IJs/ml, and aliquots were extracted and placed in containers with the following concentrations of vinasse: 0% (control group, distilled water only), 50% and 100%, kept in BOD incubators (Eletrolab®, model EL 212) at temperatures of 16 °C (control), 25 °C, and 35 °C. The containers were taken out of the incubators after 72h, and aliquots were analyzed under an optical microscope to determine the effects caused by temperature and vinasse on EPNs viability.

Seventy-two hours after the beginning of the viability bioassay, surviving nematodes from each concentration/temperature were used in a subsequent bioassay to evaluate their efficacy to stable fly larvae in Petri dishes.

Aliquots were extracted from each group and quantified at 400 viable IJs/larva of *S. calcitrans*. Six-day-old *S. calcitrans* larvae were subjected to mortality tests involving five larvae per Petri dish (nine centimeters in diameter). Petri dishes were kept on shelves in the laboratory at 27±1 °C and 70±10% RH. The experiment was conducted in an entirely randomized design with six repetitions. The bioassay was monitored daily for seven days. *S. calcitrans* larvae mortality data were subjected to the Shapiro-Wilk normality test. After detecting normality of the distribution, an analysis of variance (three-way ANOVA) was applied, and if the groups showed a significant effect, the means were compared using Tukey’s test. In all analyses, a global significance level of 5% was adopted. All the analyses were performed using Prisma GraphPad version 9.1.2 software.

### Bioassay II

#### Efficacy of EPNs on *S. calcitrans* larvae of different ages in filter cake

Groups of five 4, 6 and 8-day-old larvae were placed on 9-cm diameter Petri dishes (lined with filter paper) containing 2.5 grams of filter cake, and exposed to a concentration of 400 IJs/larva of *S. calcitrans* (diluted in 4 mL of distilled water). The volume of water added to the control groups was the same as the experimental groups, but without EPNs. The dishes were kept on shelves in the laboratory at 27±1 °C and 70±10% RH. The experiment was performed in an entirely randomized design with six repetitions. The treatment groups resulted from the 2x3 factorial design of two EPNs species (*H. bacteriophora* HP88 and *H. baujardi* LPP7) and three ages of larvae (four, six and eight days) developing in filter cake. The bioassay was monitored daily for seven days. *S. calcitrans* larvae mortality data were subjected to the Shapiro-Wilk normality test. After detecting normality of the distribution, an analysis of variance (two-way ANOVA) was applied, and if the groups showed a significant effect, the means were compared using Tukey’s test. In all analyses, a global significance level of 5% was adopted. All the analyses were performed using Prisma GraphPad version 9.1.2 software.

### Bioassay III

#### Efficacy of different concentrations of EPNs on *S. calcitrans* larvae in sugarcane bagasse

Groups of five 6-day-old larvae were placed on Petri dishes (9 cm diameter, lined with filter paper) containing three grams of chopped sugarcane bagasse, to which were added 100, 300 and 500 IJs/larva of *S. calcitrans* diluted in 4 ml of distilled water. The volume of water added to the control groups was the same as the experimental groups, but without EPNs. The plates were kept on shelves in the laboratory at 27±1 °C and 70±10% RH. The experiment was carried out in an entirely randomized design, with seven treatment groups subjected to six repetitions. Efficacy of *H. bacteriophora* HP88 and *H. baujardi* LPP7 on stable fly larvae was evaluated in sugarcane bagasse. The bioassay was monitored daily for seven days. *S. calcitrans* larvae mortality data were subjected to the Shapiro-Wilk normality test. After detecting normality of the distribution, an analysis of variance (one-way ANOVA) was applied, and if the groups showed a significant effect, the means were compared using Tukey’s test. In all analyses, a global significance level of 5% was adopted. All the analyses were performed using Prisma GraphPad version 9.1.2 software.

## Results and Discussion

### Efficacy of EPNs against *S. calcitrans* larvae

A significant effect was observed in the in the EPNs vs. vinasse and EPNs vs. temperature interactions, indicating that the lethality of the nematodes evaluated varied as a function of vinasse concentrations and storage temperature. There was no significant effect of the triple interaction of the tested factors. The EPN *H. bacteriophora* HP88 showed higher efficacy than *H. baujardi* LPP7 at all temperatures, especially at 50% and 100% vinasse, with more pronounced mortality at 35°C at 100% vinasse concentration (Table 1). When the efficacy of EPNs was evaluated as a function of increasing temperature, the virulence of *H. bacteriophora* HP88 did not decrease at all vinasse concentrations. However, the virulence of *H. baujardi* LPP7 decreased as the temperature increased, especially at concentrations of 50% and 100% vinasse (Table 1).

**Table 1:** Mortality rates of *Stomoxys calcitrans* larvae due to the entomopathogenic nematodes *Heterorhabditis bacteriophora* HP88 and *H. baujardi* LPP7 at different vinasse concentrations and storage temperatures.

Groups	*Stomoxys calcitrans* mortality rate (%)							
	EPNs vs. temperature (°C) vs. vinasse concentrations (%)							
	0%			50%			100%	
	HP88	LPP7		HP88	LPP7		HP88	LPP7
16 °C	100.0 Axa	93.3 Axa		93.3 Axa	90.0 Axa		86.7 Axa	63.3 Bya
25 °C	100.0 Axa	90.0 Axa		100.0 Axa	76.7 Bxb		86.7 Axa	56.7 Bya
35 °C	100.0 Axa	86.7 Axa		100.0 Axa	70.0 Bxb		96.7 Axa	33.3 Byb
Mean	100.0 A	90.0 A		97.8 A	78.9 B		90.0 A	51.1 B

Mean values followed by the same letter, lower case in the columns and upper case in the rows, do not differ by Tukey’s test (p > 0.05), with 'x' and 'y' comparing each EPN within the vinasse concentrations (x > y).

Vinasse had no negative effect on the virulence of *H. bacteriophora* HP88, regardless of the temperature applied, since the mortality rate of fly larvae was always greater than 86% in all concentrations of vinasse subjected to all temperatures. For *H. baujardi* LPP7, there was no statistical difference in larval mortality when this EPN was applied at 50% vinasse compared to without vinasse. However, when subjected to 100% vinasse, at all tested temperatures, this EPN decreased its virulence, since larval mortality was lower, especially at 25° and 35 °C (Table 1).

Overall, *H. bacteriophora* HP88 showed higher mortality rates than *H. baujardi* LPP7 against *S. calcitrans* larvae, and its virulence was not affected by increasing of temperature and vinasse concentration. Conversely, the virulence of *H. baujardi* LPP7 declined with increasing of temperature and vinasse concentrations, indicating that this EPN is more sensitive to these conditions than *H. bacteriophora* HP88.

The conditions of this bioassay provided some of the most important information of the data set obtained from the present study, because, considering the possibility of creating an integrated stable fly control system using EPNs, the temperature that these organisms can withstand is a crucial factor for the success of such an application. These data indicate that IJs of *H. baujardi* LPP7 are highly vulnerable to both pure vinasse and temperatures close to the limit for stable fly development (Florez-Cuadros et al., 2019), while *H. bacteriophora* HP88 proved to be little affected. Studies dealing with the heat resistance of the genus *Heterorhabditis* indicate that, in general, the optimal temperature for the development, infection, and reproduction of these EPNs is around 25 °C (Shaurub et al., 2015). However, several publications reported the adaptation of EPNs species to temperatures around 35 °C (Kusakabe et al., 2019). Similarly, the considerable efficacy of EPNs for pest control has been confirmed even when exposed to high temperatures, and on a wide variety of hosts (Mendonça et al., 2019). In the specific case of *H. baujardi* LPP7, an optimal temperature of 28 °C for replication and infection has been reported, with emphasis on the origin of this strain, namely, the Amazon rainforest; hence, it is probably already adapted to tropical environments (Dolinski et al., 2007). González (2006) reported the viability of *H. baujardi* LPP7 at temperatures of 35 °C for two hours, with no loss in efficacy against *G. mellonella* larvae. In the present study, *H. baujardi* LPP7 lost efficacy against *S. calcitrans* larvae after 72 h (in a different substrate) at 35°C. Furthermore, it should be noted that this EPN has been evaluated on different hosts and that *G. mellonella* showed considerably greater sensitivity to this EPN than the stable fly.

The resistance of these EPNs to solutions containing 50% vinasse, and the short time required to reach high mortality peaks at this concentration, suggest that EPNs could be used in combination with the fertigation of sugarcane fields with vinasse, since this is the vinasse concentration usually applied (Rossetto, 1987). Corrêa et al. (2013) described the storage of vinasse in pre-fertigation tanks, which may also be an approach for the application of EPNs, or even the addition of EPNs in hydrants near sprinklers, where the temperature should be even lower, and occasionally in places previously identified with high numbers of *S. calcitrans* larvae. It should be noted that despite the possible high productivity (flies/area) in filter cake, the size of areas under fertigation is a more significant factor (Corrêa et al., 2013). Therefore, vinasse plus mulch is the most important substrate associated with the occurrence of *S. calcitrans* outbreaks (Corrêa et al., 2013), since it attracts stable flies and hastens the process of sugarcane straw decomposition (Souza et al., 2021).

### Efficacy of EPNs on *S. calcitrans* larvae of different ages in filter cake

The age of stable fly larvae did not affect their mortality rates due to EPNs. Both *H. bacteriophora* HP88 and *H. baujardi* LPP7 showed higher larvae mortality than the control group (Table 2).

**Table 2:** Mortality rates of *Stomoxys calcitrans* larvae of different ages due to the entomopathogenic nematodes *Heterorhabditis bacteriophora* HP88 and *H. baujardi* LPP7 in filter cake.

	*S. calcitrans* mortality rates (%)			
	Age of *S. calcitrans* larvae (in days)			
EPN	4d	6d	8d	Mean
Control	3.33 Ab	0.00 Ab	0.00 Ac	1.11 c
HP88	90.00 Aa	80.00 Aa	83.33 Aa	84.44 a
LPP7	80.00 Aa	60.00 Ba	53.33 Bb	64.44 b
Mean	57.78 A	46.67 A	45.56 A	

Mean values followed by the same letter, upper case in rows and lower case in columns, do not differ by Tukey's test (p<0.05).

Not many experiments are reported in the literature regarding the interaction between filter cake and insect pests that develop in this substrate. Monteiro et al. (2016), using a similar methodology with lower concentrations of *H. bacteriophora* HP88, i.e, 25, 50, 100, 150 and 200 IJs/larva of *S. calcitrans* at eight days of age. The larvae mortality rate reported by the above-mentioned authors was 76.7% with 150 IJs/larva and 83.3% when applying 200 IJs/larva. The high mortality of stable fly larvae of different ages by *H. bacteriophora* HP88 in the present study agrees with the previous findings of Monteiro et al. (2016).

The larvae mortality rate achieved in the present study with *H. bacteriophora* HP88 was lower (83.33%) than the 96.7% mortality reported by Leal et al. (2017) using 200 IJs/larva. However, in the bioassay developed by the cited authors, EPNs were not exposed to substrates that could influence their effectiveness. Hence, it is reasonable to infer that filter cake had a limited impact on the action of *H. bacteriophora* HP88 in the present study. This byproduct of the sugar and ethanol industry may interfere more significantly in the virulence of *H. baujardi* LPP7, since Leal et al. (2017) reported that the proportion of 200 IJs/larva of *S. calcitrans* succeeded in causing a 93.3% mortality rate.

An important factor for the occurrence of population outbreaks of this fly in Brazil is the huge volume of byproducts generated by the sugarcane industry and the large quantities left behind on the field after harvesting. Therefore, it is worth considering that the area needed for the application of EPNs in filter cake under natural conditions would be drastically smaller if compared to the area of straw that remains on the ground after the sugarcane harvest, since the filter cake is stored in windrows (a more limited area). Furthermore, the heat produced by filter cake fermentation reaches 35.5 ± 5.8°C (Corrêa et al., 2013) limiting distribution of stable fly larvae to windrow edges. These areas are less hot and more humid, also facilitating the movement of EPNs in their search for stable fly larvae.

### Efficacy of different concentrations of EPNs on *S. calcitrans* larvae in sugarcane bagasse

**Figure 1:**
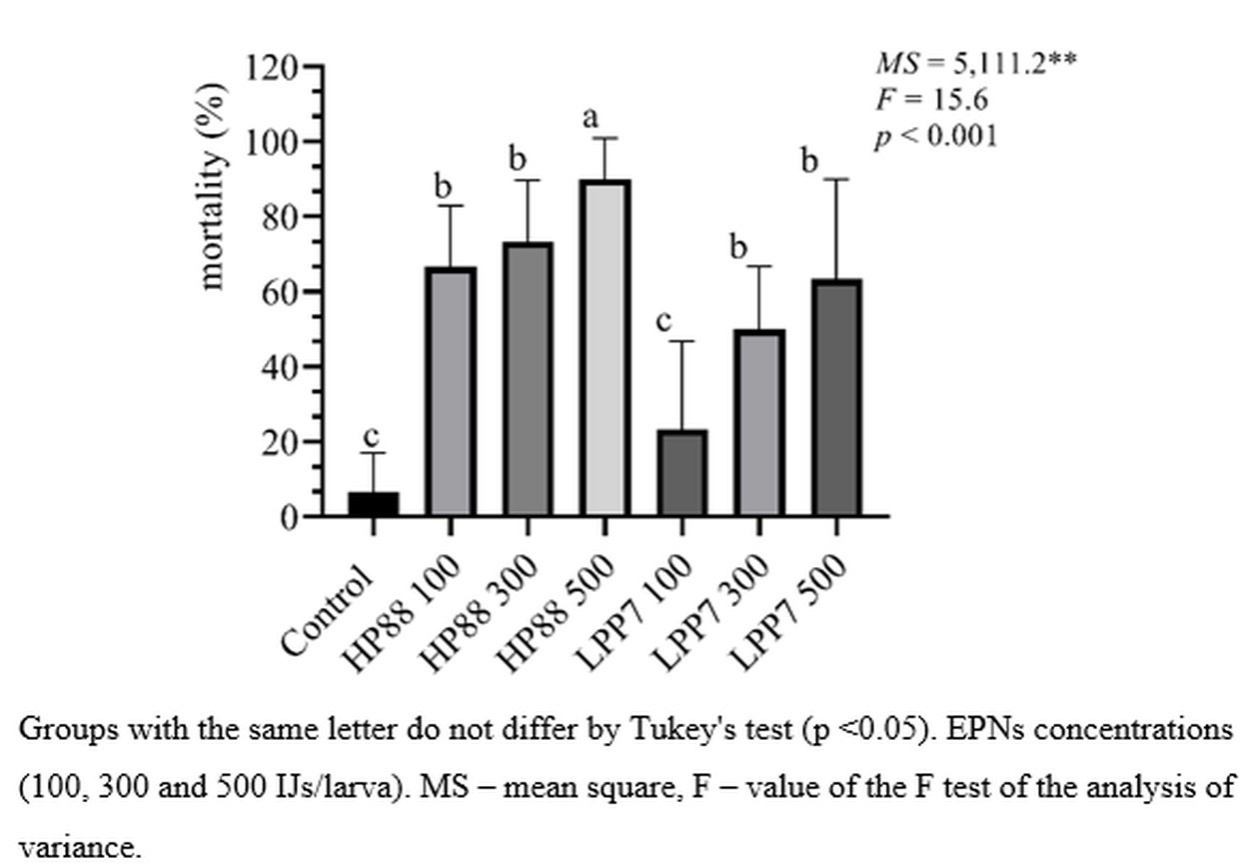
Mortality rates of *Stomoxys calcitrans* larvae exposed to different concentrations of the entomopathogenic nematodes *Heterorhabditis bacteriophora* HP88 and *H. baujardi* LPP7 in sugarcane bagasse.

The EPN *H. bacteriophora* HP88 caused higher mortality rates than the control group at all the tested concentrations. The concentration that caused the highest mean mortality rate was 500 IJs/larva, which was significantly higher than the concentrations of 100 and 300 IJs/larva; the latter showed no statistically significant difference (Figure 1). It was noted that the more than 60% mortality rate of larvae treated with 500 IJs of *H. baujardi* LPP7, which is higher than that obtained in other biological pest control studies (Moraes et al., 2008; Alves et al., 2012). Of particular importance for control systems using fungi is that certain antifungal and antibacterial substances are released by the larval microbiota of *S*. *calcitrans* (Dillon & Dillon, 2004; Moraes et al., 2014). Given that the pathogenicity of EPNs is caused by septicemia after invasion and secretion of bacteria directly inside the host, it is possible that these nematode species are not directly affected by the larval microbiota of the dipteran, which explains the difference commonly reported in the mortality rates of immature stages of *S. calcitrans* caused by these two control methods.

A comparison of the results of this bioassay with the findings reported by Leal et al. (2017) using the same concentration of 100 IJs/larva revealed a loss of efficacy of *H. bacteriophora* HP88 in the order of 30%. In the case of *H. baujardi* LPP7, this comparison reveals a lower mortality rate of 53.4%, with the caveat that the method employed by Leal et al. (2017) did not use materials such as sugarcane on the infection dishes, indicating that the decrease in treatment efficacy (100 IJs/larva) is likely associated with the presence of sugarcane bagasse.

## Conclusions

The results found in the present study suggest that EPNs can be a potential component in integrated strategies of stable fly control and outbreak prevention in areas of sugar and alcohol production.
